# SeroTools: a Python package for *Salmonella* serotype data analysis

**DOI:** 10.21105/joss.02556

**Published:** 2020-09-05

**Authors:** Joseph D. Baugher

**Affiliations:** 1 Center for Food Safety and Applied Nutrition, U.S. Food and Drug Administration

## Summary

Subtyping, the ability to differentiate and characterize closely related microorganisms, has historically been a critical component of successful outbreak identification and traceback efforts employed by public health researchers and regulatory agencies for foodborne pathogens. Serological subtyping (or serotyping) has been the standard approach, largely based on antibody binding to surface antigens (Henriksen, 1978). The identification of specific antigenic factors has facilitated the creation of serotyping schemes, which define each serovar using a specific (generally unique) combination of antigenic factors. Serotyping schemes have been developed to assist in characterization of many microorganisms, including pathogens such as *Salmonella*, *E. coli*, *Shigella* (Strockbine, Bopp, Fields, Kaper, & Nataro, 2015), *Streptococcus* (Spellerberg & Brandt, 2015), and *H. influenzae* (Ledeboer & Doern, 2015).

*Salmonella* is a major foodborne pathogen for which serotyping has played a fundamental monitoring role for over 50 years(CDC, n.d.). *Salmonella* serotyping is generally based on antibody binding to the O antigen (a surface antigen) and one or more H antigen phases (flagellar antigens) (Andrews, Wang, Jacobson, & Hammack, 2019; Strockbine et al., 2015). The White-Kauffmann-Le Minor (WKL) *Salmonella* scheme specifies the naming and formatting conventions for *Salmonella* serotyping data and the antigenic factors (and other characteristics) which define each serovar (Grimont & Weill, 2007). SeroTools includes the 2007 WKL scheme (Grimont & Weill, 2007) and updates (Bugarel et al., 2015; Guibourdenche et al., 2010; Issenhuth-Jeanjean et al., 2014).

**Figure 1:**
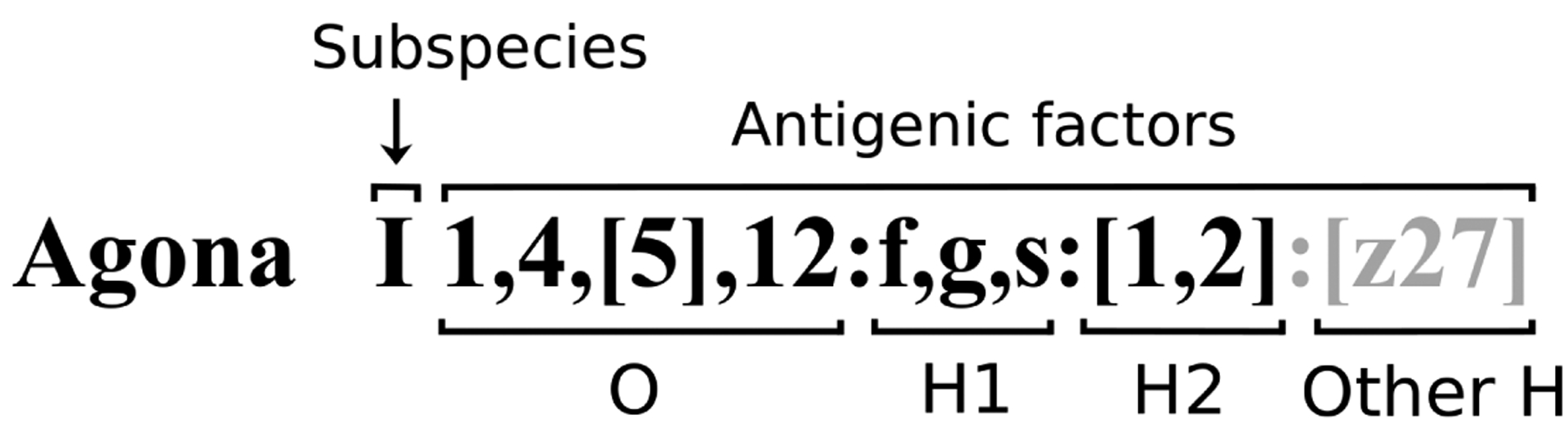
Standard formatting of the antigenic formula.

The WKL scheme currently recognizes two species of *Salmonella*, *S. enterica* and *S. bongori*. *S. enterica* is comprised of six subspecies (subsp.): *enterica* (I), *salamae* (II), *arizonae* (IIIa), *diarizonae* (IIIb), *houtenae* (IV) and *indica* (VI). Note that *S. bongori* is still frequently designated as subsp. V for scheme consistency, although it is no longer considered a subspecies of *S. enterica*. The WKL scheme assigns a unique name (e.g. serovar Enteritidis) to each of the serovars of *S. enterica* subsp. *enterica* (I), while the serovars representing the other subspecies are referred to by their antigenic formulae. The antigenic formula formatting is defined by the WKL scheme and is demonstrated for serovar Agona in Figure 1. The formula contains a subspecies designation and a colon-separated list of antigenic factors for which the following fields are required: O antigen, phase 1 H antigen, and phase 2 H antigen. The field for ‘Other H’ antigen includes R phases and third phases and is present only when populated. An antigenic formula may include additional annotation such as:

1. *Square* brackets to indicate optional factors, (e.g. I 1,4,**[5]**,12:f,g,s:**[1,2]**:**[z27]**,**[z45]**).
2. *Underlining* to indicate O factors present only in the presence of the converting phage, represented here and in SeroTools as optional (with *square* brackets) due to the inability to capture typographical formatting in plain text, (e.g. I **[1]**,9,12:e,h:1,5).
3. *Curly* brackets to indicate mutually exclusive factors, (e.g. I 3,**{10}{[15]}**:k:1,5).
4. *Parentheses* to indicate factors which are weakly agglutinable, (e.g. IIIb **(6)**,14:k:z53).
5. A *dash* to indicate a missing antigen, (e.g. I 1,9,12:g,m:**–**).

These additional annotations are captured in the SeroTools repository and employed for determination of congruence between serovars.

## Statement of Need

SeroTools addresses multiple critical needs for the efficient analysis of *Salmonella* serotyping data within the public health community. In recent years, significant technological advances have resulted in a wide range of molecular-based subtyping options, including highly sensitive approaches based on whole genome sequencing (WGS). One such approach involves the application of software tools to WGS data for *in silico* serovar prediction (Joensen, Tetzschner, Iguchi, Aarestrup, & Scheutz, 2015; Laing, Bessonov, Sung, & La Rose, n.d.; Watts & Holt, 2019; Wu, Lau, Lee, Lau, & Payne, 2019; Zhang et al., 2019, 2015), including real-time prediction (Feng et al., 2020). SeqSero (a *Salmonella*-specific tool) and other *in silico* serovar designation tools have been adopted by U.S. public health agencies as an alternative to serological testing and for quality control applications (Dowdy, 2017; Timme, Sanchez Leon, & Allard, 2019). The advent of new methodologies for serovar determination has engendered a need for method-comparison studies, and has sparked a growing collection of recent publications comparing various laboratory-based and *in silico* serovar predictions (Banerji, Simon, Tille, Fruth, & Flieger, 2020; Cooper et al., 2020; Diep et al., 2019; Ibrahim & Morin, 2018; Tang et al., 2019; Yachison et al., 2017; Zhang et al., 2019, 2015). In light of the growing interest in *in silico* serovar prediction and serotyping method-comparison studies, SeroTools provides unique tools which fill multiple gaps in the analysis process. It serves as the only multiformat WKL repository accessible for software development. Currently the WKL scheme is available only as a pdf document (Grimont & Weill, 2007) and as Python lists in SeqSero (Zhang et al., 2015) and SeqSero2 (Zhang et al., 2019). SeroTools also provides the only existing tools for querying the WKL scheme, comparing serovars for congruence, and predicting the most abundant serovar for clusters of isolates.

## Functionality and Features

The SeroTools Python package provides the following functionality:

1. Repository –
  - SeroTools includes an updated WKL repository in multiple formats, including Python data structures (a pandas DataFrame, dictionaries, and lists) and spreadsheets (Excel and tab-delimited). The repository includes fields representing serovar name, antigenic formula, species, subspecies, O antigen, phase 1 H antigen, phase 2 H antigen, other H antigens, the new O group designation (e.g. O:2), and the old O group designation (e.g. A).
2. Toolkit –
  - **query** - SeroTools provides the ability to easily query the WKL repository with serovar names or antigenic formulas.
  - **compare** - SeroTools provides a convenient method for automated comparison of serovar designations, including increased differentiation for levels of congruence.
  - **cluster** - SeroTools includes methods for robust determination of the most abundant serovar for a cluster of isolates.
3. Additional functionality –
  - SeroTools includes Pythonic data structures and a host of utility functions for analyzing and manipulating large *Salmonella* serovar datasets. Other functionality includes the ability to determine the antigenic factors common to a group of serovars.

SeroTools defines four levels of congruence for use in querying the repository and comparing serovars. Note - *optional* factors as referenced below include optional, exclusive, and weakly agglutinable factors, as specified in the WKL scheme.

1. **Exact** matches must meet **one** of the following criteria:
  - The serovar designations are the identical string.
    For example:
    CorvallisCorvallisI 8,[20]:z4,z23:[z6]I 8,[20]:z4,z23:[z6]
  - Every antigenic factor (*required* or *optional*) matches.
    For example:
    CorvallisI 8,[20]:z4,z23:[z6]I 8,[20]:z4,z23:[z6]I 8,20:z4,z23:z6I 1,3,10,19:f,g,t:1,(2),7I 1,3,10,19:f,g,t:1,2,7
  - The subspecies designations are identical and neither serovar designation includes any antigenic factors.
    For example:
    I ::I –:–:–II :II –:
2. **Congruent** matches must meet **all** of the following criteria:
  - The subspecies field must be present either for both serovars or for neither.
  - All *required* antigenic factors match.
  - Any differences are due to the presence/absence of *optional* factors.
    For example:
    I 6,7,14:g,m,s:–I 6,7,[14],[54]:g,m,[p],s:–I 6,7:g,m,s:–I 6,7,[14],[54]:g,m,[p],s:[1,2,7]Amager var. 15+AmagerI 3,15:y:1,2:[z45]I 3,{10}{15}:y:1,2:[z45]6,7:k:[z6]6,7:k:–
3. **Minimally congruent** matches must meet the following criteria:
  - Every antigen of at least one serovar can be considered a formal subset of the corresponding antigen (no direct conflicts). Note - the empty set (–) is a subset of every set.
    For example:
    I 6,7,14,[54]:g,m,[p],s:–6,7,[14],[54]:g,m,[p],s:–II 6,7,8,[14],[54]:g,m,[p],s:–I 7:g:–I 6,7:g,m,s:–GallinarumEnteritidis
4. **Incongruent** matches must meet the following criteria:
  - Any comparison which is not at least minimally congruent.
    For example:
    IIII 1:1 2:JavianaSaintpaulI 7,8:g,m,s:–I 6,7,[14],[54]:g,m,[p],s:[1,2,7]I 4,5:a,b:6,7I 5:a,b,c:6,7

The ‘minimally congruent’ designation is unique to SeroTools and is useful for distinguishing between two scenarios: serovars which differ due to sample misannotation (truly incongruent) and serovars derived from correctly annotated samples with variation based solely on missing information. When comparing serovar predictions, minor differences may be expected due to method-specific irregularities, for example, reagent variation for laboratory-based techniques or sequencing read coverage for *in silico* techniques. Our assumption is that these minor method-specific differences are more likely manifested as missing data (e.g. all but one of the correct factors were detected) than direct conflicts.

## Links

Documentation: https://serotools.readthedocs.io/en/latest/readme.html

Source Code: https://github.com/CFSAN-Biostatistics/serotools

PyPI Distribution: https://pypi.python.org/pypi/serotools
